# PI3K couples long-term synaptic potentiation with cofilin recruitment and actin polymerization in dendritic spines via its regulatory subunit p85α

**DOI:** 10.1007/s00018-024-05394-x

**Published:** 2024-08-19

**Authors:** Sergio López-García, Esperanza López-Merino, Alba Fernández-Rodrigo, Pablo Zamorano-González, Silvia Gutiérrez-Eisman, Raquel Jiménez-Sánchez, José A. Esteban

**Affiliations:** 1 Department of Molecular Neuropathology, Centro de Biología Molecular Severo Ochoa (CSIC-UAM), Madrid, Spain; 2 Current address: Universidad de Málaga, Málaga, Spain

**Keywords:** p85, Rac1, Structural plasticity, Spine, LTP, LTD

## Abstract

Long-term synaptic plasticity is typically associated with morphological changes in synaptic connections. However, the molecular mechanisms coupling functional and structural aspects of synaptic plasticity are still poorly defined. The catalytic activity of type I phosphoinositide-3-kinase (PI3K) is required for specific forms of synaptic plasticity, such as NMDA receptor-dependent long-term potentiation (LTP) and mGluR-dependent long-term depression (LTD). On the other hand, PI3K signaling has been linked to neuronal growth and synapse formation. Consequently, PI3Ks are promising candidates to coordinate changes in synaptic strength with structural remodeling of synapses. To investigate this issue, we targeted individual regulatory subunits of type I PI3Ks in hippocampal neurons and employed a combination of electrophysiological, biochemical and imaging techniques to assess their role in synaptic plasticity. We found that a particular regulatory isoform, p85α, is selectively required for LTP. This specificity is based on its BH domain, which engages the small GTPases Rac1 and Cdc42, critical regulators of the actin cytoskeleton. Moreover, cofilin, a key regulator of actin dynamics that accumulates in dendritic spines after LTP induction, failed to do so in the absence of p85α or when its BH domain was overexpressed as a dominant negative construct. Finally, in agreement with this convergence on actin regulatory mechanisms, the presence of p85α in the PI3K complex determined the extent of actin polymerization in dendritic spines during LTP. Therefore, this study reveals a molecular mechanism linking structural and functional synaptic plasticity through the coordinate action of PI3K catalytic activity and a specific isoform of the regulatory subunits.

**Supplementary Information:**

The online version contains supplementary material available at 10.1007/s00018-024-05394-x.

## Introduction

Synaptic plasticity refers to the activity-dependent modifications of synapses that alter the strength or efficacy of synaptic transmission. This property is widely accepted to underlie learning and memory processes [1–3]. Functional changes in synaptic transmission are often accompanied by structural changes of dendritic spines and/or remodeling of synaptic connectivity [4–7]. However, the underlying molecular mechanisms that link and coordinate functional and structural aspects of synaptic plasticity are far from straightforward and remain poorly understood.

Within the functional aspects of synaptic plasticity, long-term potentiation (LTP) and long-term depression (LTD) at CA1 hippocampal synapses rely on the regulated trafficking of AMPA-type glutamate receptors (AMPARs) into and from the post-synaptic membrane, as well as on post-translational modifications [8–13]. Structural plasticity is mediated by the remodeling of the actin cytoskeleton of the dendritic spine through the engagement of actin-binding proteins (ABPs), which leads to enlargement or shrinkage of existing spines, together with the formation or retraction of spines [4, 14–19]. Key regulators of the actin cytoskeleton dynamics are the actin-depolymerizing factor (ADF)/cofilin proteins [20]. Cofilin is rapidly and persistently enriched within spines upon LTP induction [21–23], where it promotes actin filament turnover through its severing activity [24–26].

In terms of signaling pathways, class I phosphatidylinositol 3-kinases (PI3Ks) [27] are known to drive functional changes in AMPAR-mediated synaptic transmission during plasticity [28–35]. On the other hand, PI3K signaling has been associated with neuronal growth and synaptogenesis [35–38]. Therefore, these PI3Ks are well suited to coordinate structural and functional aspects of synaptic plasticity, although how this coordination may occur is still unknown. Class IA PI3Ks are heterodimeric proteins, where the catalytic subunit (p110) forms an obligatory complex with a regulatory subunit (p85α, p85β, p55α, p50α or p55γ). Using genetic approaches, we have recently shown that different isoforms of the catalytic subunit are specialized for distinct forms of synaptic plasticity and structural maintenance of hippocampal synapses [35]. In contrast, the role of the regulatory subunits in the brain is much more uncertain. Interestingly, p85 subunits have been linked to small GTPases of the Rho family, particularly Rac1 and Cdc42, which are known regulators of the actin cytoskeleton [39–41]. This pathway is in turn connected with cofilin regulation and synaptic function via LIM kinase [21, 42–44]. On the other hand, the relevance of p85 subunits for brain function is supported by the behavioral and cognitive deficits observed in p85α-deficient mice [45]. However, the pleiotropic phenotypes of these animals (extensive loss of synapses and myelinated axons [46]) make it difficult to establish specific functions for these subunits at synapses.

In this study, we hypothesized that the regulatory subunits of PI3K provide additional functionalities in relation to the mechanisms of synaptic plasticity. To address this hypothesis, we have targeted specific p85 isoforms using shRNA knock-down. This strategy allows a semi-acute gene inactivation, which may avoid the pleiotropic effects of the prolonged elimination of these proteins. Using this approach, we have found that p85α (and not p85β) is absolutely required for NMDA receptor (NMDAR)-dependent LTP, but not for basal synaptic transmission or mGluR-dependent LTD. This specificity for p85α was also expressed at the level of structural plasticity, in terms of cofilin recruitment and actin polymerization in spines after LTP induction. Thus, our findings reveal a distinct contribution of PI3K regulatory subunits to synaptic plasticity and provide a molecular mechanism to link structural and functional synaptic plasticity.

## Results

### p85α, but not p85β, is required specifically for LTP

To start evaluating the requirement for the different p85 regulatory subunits in synaptic plasticity, we took advantage of lentiviral-based shRNA expression to selectively knock-down p85α or p85β in rat hippocampal neurons. In the case of the p85α mRNA, two different shRNAs were designed: (i) shp85α (L), which targets specifically the long (p85α) isoform, and (ii) shp85α (L + S), which knocks-down the long (p85α) and the short (p55α/p50α) regulatory isoforms, encoded by the *PIK3R1* gene by alternative splicing [47–49]. Another shRNA was designed against the mRNA sequence of p85β (shp85β) (see Methods for the targeting sequences and Suppl. Figure 1A for domain organization and PI3K subunit associations). The efficacy and specificity of these shRNAs to knock-down their respective targets was confirmed on dissociated rat hippocampal neurons infected with the different lentiviral vectors (Suppl. Figure 1B). To note, the shRNAs for p85α produced a significant increase in p85β expression levels, particularly when all p85α-related subunits were removed (shp85α (L + S)), perhaps as a compensatory effect. Importantly, none of these shRNAs altered the expression levels of p110α and p110β catalytic subunits (Suppl. Figure 1B). Instead, the subunit composition of the PI3K heterodimers was shifted, by driving the association of the p110 catalytic subunit with the remaining p85 subunit (p85α in the case of the shp85β, and p85β in the case of the shp85α (L + S); Suppl. Figure 1C). Therefore, this strategy allows us to specifically remove p110/p85α or p110/p85β complexes without globally altering p110 levels or p110/p85 heterodimerization.

**Fig. 1:**
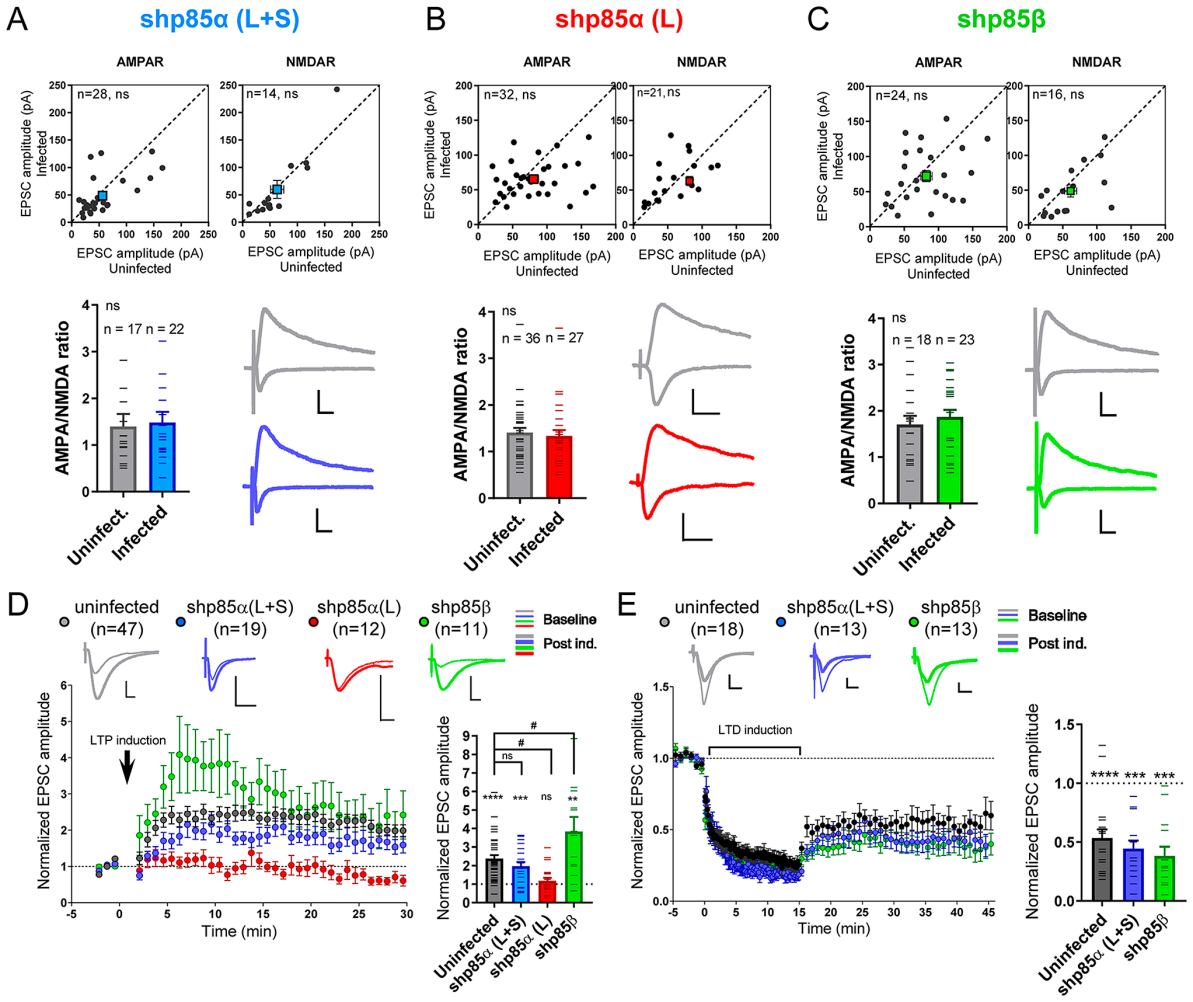
Effect of p85 shRNAs on synaptic function and plasticity. **A-C.** Whole-cell voltage-clamp recordings of CA3-to-CA1 synaptic responses mediated by AMPARs (upper left panels) or NMDARs (upper right panels) from uninfected neurons (control) or from neighboring neurons infected with lentiviral vectors for the expression of shp85α (L + S) (**A**), shp85α (L) (**B**) or shp85β (**C**). EPSC amplitudes for each pair of cells are represented as black circles in the scatter plots. Average responses are indicated with colored squares (mean ± SEM). The ratio of AMPAR to NMDAR responses is also plotted from individual cells (lower left panels). Representative traces from uninfected and infected neurons are shown in the lower right panels. Scale bars represent 50 pA (Y axis), 20 ms (X axis). Statistical differences between conditions were assessed by Wilcoxon t-test (paired recordings) or by Mann-Whitney test (AMPA/NMDA ratios). n: number of cells. ns: not significant. **D**,** E. ***Left*, time course of the normalized AMPAR-mediated responses (mean ± SEM) during a baseline period and after induction of NMDAR-dependent LTP (**D**) or mGluR-dependent LTD (**E**). EPSC amplitude is normalized to the average baseline. *Right*, histogram showing EPSC amplitude for each cell collected from the first 5–10 min of the recording after induction of NMDAR-dependent LTP (**D**) or the last 5 min of the recording after induction of mGluR-dependent LTD (**E**), normalized to their average baseline. Individual values for each experiment are represented as lines overlying the columns. Bars show mean ± SEM. Significant potentiation or depression was assessed via Wilcoxon t-test. ****p < 0.0001, ***p < 0.001, **p < 0.01. Significant differences between conditions were evaluated by one-way ANOVA test followed by the Tukey’s multiple comparisons test. #p < = 0.05. n: number of cells. ns: not significant. Representative traces are shown above the time courses for baseline (thin lines) and post-induction (thick lines). Scale bars represent 50 pA (Y axis), 10 ms (X axis)

Once verified the efficiency and specificity of the shRNAs, we analyzed their effect on synaptic function using organotypic hippocampal slice cultures. The different shRNAs were expressed via lentiviral infection for 7 days in CA1 neurons, and then we carried out simultaneous recordings of AMPAR- and NMDAR-mediated currents from neighboring infected and uninfected neurons while stimulating the Schaffer collateral afferents. As shown in Fig. 1A-C, none of these shRNAs altered basal synaptic transmission mediated by AMPARs or NMDARs, indicating that the identity of the regulatory PI3K subunit (p85α/p55α/p50α or p85β) is not relevant for basal excitatory CA3-to-CA1 synaptic transmission.

We then evaluated the contribution of p85α/p55α/p50α and p85β to the two forms of synaptic plasticity in which PI3K has been shown to be involved, namely NMDAR-dependent LTP and mGluR-dependent LTD [28–31, 35]. Interestingly, shp85α (L) and shp85β produced virtually opposite results with respect to synaptic potentiation (Fig. 1D). LTP was essentially abolished when only p85α was removed (shp85α (L)), that is, in the presence of p85β and the small isoforms p55α/p50α (Fig. 1D, red symbols). Conversely, there was a transient, but statistically significant increase in potentiation, as compared with uninfected neurons, when p85β was removed (Fig. 1D, green symbols). These results suggest divergent roles for p85α and p85β in LTP, with p85α being most critically required for synaptic potentiation. Intriguingly, the effect on LTP was intermediate and not significantly different from control neurons when p85α/p55α/p50α levels were simultaneously reduced (shp85α (L + S)), although there was a tendency towards reduced potentiation (Fig. 1D, blue symbols). On this point, it is worth noting that shp85α (L + S) produced a marked (about 3-fold) overexpression of p85β (Suppl. Figure 1B), which may be responsible for a partially compensatory effect on LTP.

In the case of mGluR-LTD, we found no significant changes between uninfected control and shp85α (L + S)- or shp85β-expressing neurons (Fig. 1E). These results suggest that the identity of the regulatory isoform is not relevant for mGluR-LTD on CA1 neurons.

Finally, passive membrane properties of the cell (whole-cell membrane capacitance and input resistance) were not altered in shp85α (L + S)-, shp85α (L)- or shp85β-infected neurons, as compared to uninfected controls (Suppl. Figure 2A).

### Activity-dependent cofilin recruitment into spines is mediated by the regulatory subunit p85α

Once identified p85α as an important requirement for LTP (with the potential modulation of early LTP by p85β), we then explored possible mechanisms for the participation of these regulatory subunits in synaptic plasticity. Specifically, we have addressed the potential role of p85α and/or p85β in structural plasticity, as these regulatory subunits have been linked to small GTPases of the Rho family that are known regulators of the actin cytoskeleton [39–41]. Cofilin is a critical regulator of actin dynamics at synapses [44], which gets recruited into spines very rapidly upon LTP induction [21]. Therefore, we decided to evaluate the potential role of the p85 regulatory subunits in this behavior.

**Fig. 2:**
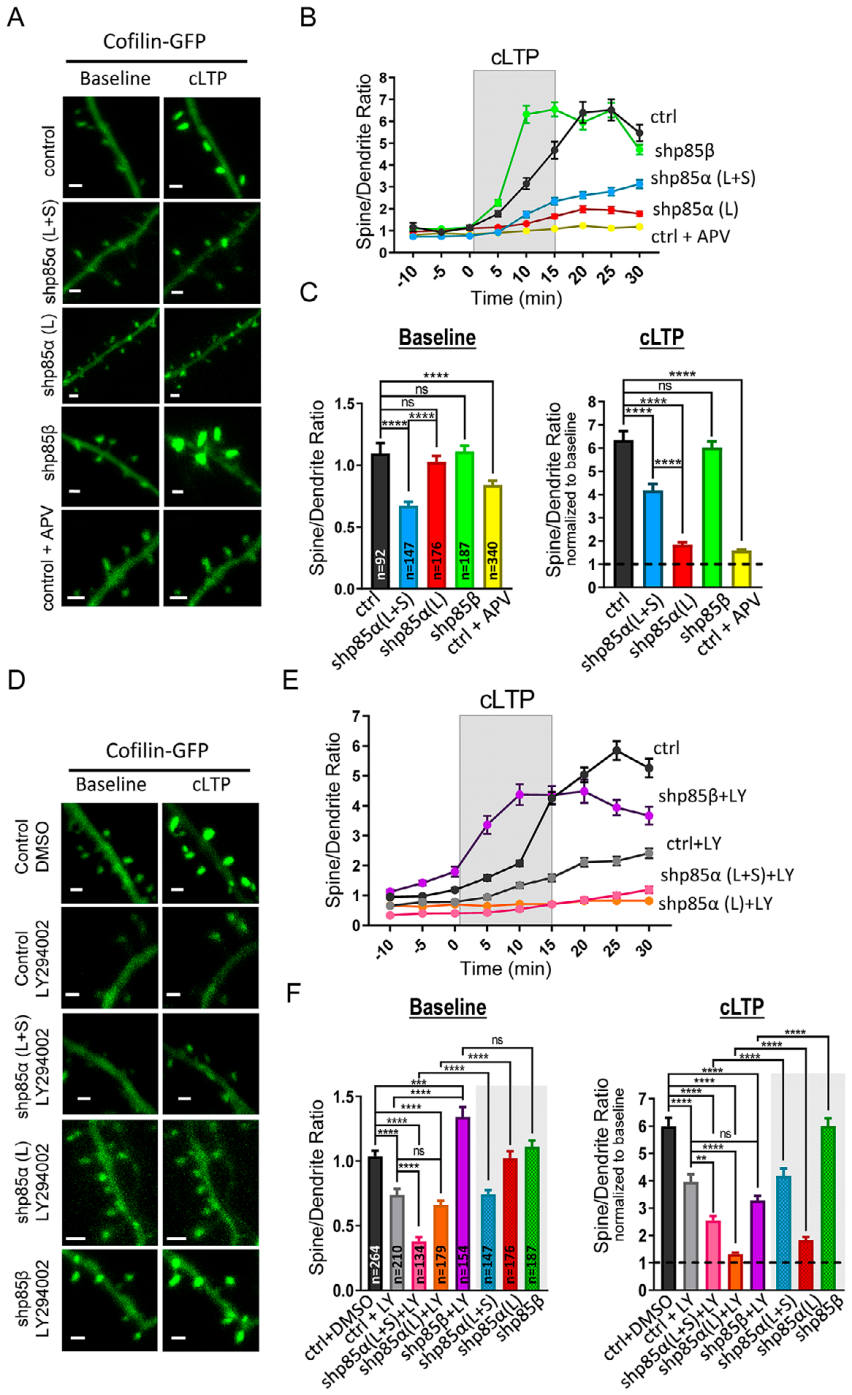
Effect of p85 shRNAs and PI3K activity on GFP-cofilin recruitment into spines during cLTP. **(A)** Representative images of dendritic branches from CA1 hippocampal neurons expressing GFP-cofilin together with shp85α (L + S), shp85α (L), shp85β or an empty vector (EV, control). Some slices expressing the empty vector were treated with 100 µM APV (NMDAR antagonist). Images are shown for baseline and after cLTP induction (15–25 min). Scale bar: 1 μm. **(B)** Quantification of GFP fluorescence signal in spines relative to the adjacent dendritic shaft (spine/dendrite ratio) before, during (grey rectangle) and after cLTP induction, from neurons expressing shp85α (L + S) (blue symbols), shp85α (L) (red symbols), shp85β (green symbols) or the empty vector control (black symbols). Some slices expressing GFP-cofilin and the empty vector were treated with 100 µM APV 1 h prior and during the experiment (yellow symbols). Plot represents mean ± SEM. **(C)** Histogram plot of spine/dendrite ratios from baseline (*left*; -10–0 min) and after cLTP induction (*right*; 15–25 min, normalized to baseline values for each condition). Bars show mean ± SEM. n represents number of spines from 3–6 independent experiments in each condition. Statistical differences between conditions were evaluated through Kruskal-Wallis and Mann-Whitney U tests. ****p < 0.0001, ns: not significant. **(D)** Similar to A, with slices treated with LY294002 (PI3K inhibitor) or the vehicle control (DMSO). **(E)** Similar to B, with slices treated with 10 µM LY294002 and expressing shp85α (L + S) (pink symbols), shp85α (L) (orange symbols), shp85β (magenta symbols) or the empty vector control (grey symbols). Slices treated with the vehicle control (DMSO) and expressing the empty vector are represented with black symbols. Treatments were for 1 h prior imaging and during the imaging session. Plots represent mean ± SEM. **(F)** Similar to C, from the values represented in E. Values from neurons expressing shp85α (L + S) (blue symbols), shp85α (L) (red symbols), and shp85β (green symbols) without LY294002 incubation are replotted from panel C, for comparison. Bars show mean ± SEM. n represents number of spines from 4–5 independent experiments in each condition. Statistical differences between conditions were evaluated through Kruskal-Wallis and Mann-Whitney U tests. ****p < 0.0001, ***p < 0.001, **p < 0.01, ns: not significant

To this aim, organotypic hippocampal slices were biollistically cotransfected with GFP-tagged cofilin and with the shRNAs for p85α or p85β (or an empty vector as control). The shRNA plasmids and the empty vector also express mCherry, which serves to confirm cotransfection. We performed live-cell imaging experiments of transfected CA1 hippocampal neurons while inducing chemical LTP (cLTP), as a pharmacological approach to maximize the number of synapses undergoing plasticity [50] (see representative images in Fig. 2A). GFP fluorescence signal was quantified from spine heads and the adjacent dendritic shaft, and expressed as spine/dendrite ratio. This approach normalizes for different expression levels across neurons. As shown in Fig. 2B, C (“Baseline” panel; black symbols), cofilin is not particularly concentrated in spines in the basal state (spine/dendrite ratio ≈ 1). However, after induction of cLTP, cofilin is very strongly recruited into spines (Fig. 2B, C; “cLTP” panel; black symbols), in agreement with previous reports [21]. This activity-induced recruitment was dependent on NMDAR activation, as it was blocked by APV (Fig. 2B, C; yellow symbols). The specific removal of the long p85α isoform (shp85α (L)) almost fully ablated the spine recruitment of cofilin induced by cLTP, without altering its basal levels (Fig. 2B, C, red symbols). In marked contrast, the removal of p85β (shp85β), produced an accelerated cLTP-dependent cofilin recruitment compared to the control situation, again, without changes in basal concentration (Fig. 2B, C, green symbols). On the other hand, we did observe a significant reduction in the basal concentration of cofilin in spines when removing long and short p85α-related isoforms (shp85α (L + S); Fig. 2B, C; “Baseline” panel, blue symbols). Interestingly, under these conditions, cLTP-induced cofilin recruitment was also impaired (Fig. 2B, C; “cLTP” panel, blue symbols), although to a lesser extent that upon removal of only p85α (shp85α (L)) (Fig. 2B, C, compare blue and red symbols).

Therefore, these data indicate that the efficient recruitment of cofilin into spines after cLTP induction specifically requires p85α, and is accelerated in the absence of p85β. Importantly, these effects are very parallel to those observed for synaptic potentiation in LTP experiments (Fig. 1D).

In order to evaluate the potential interaction between p85 and PI3K catalytic activity, we decided to interfere with these two factors separately or in combination (see representative images in Fig. 2D). To test the specific role of PI3K catalytic activity, transfected hippocampal slices were incubated with 10 µM LY294002 (PI3K inhibitor) or with the vehicle control (DMSO) (LY294002 and DMSO were added to the slice culture medium one hour before the imaging experiment, and were also present in the ACSF during the imaging session). As shown in Fig. 2E, F (“Baseline” panel), blockade of PI3K catalytic activity produced a significant reduction in the basal accumulation of GFP-cofilin in spines (compare black and grey symbols). In addition, cLTP-induced recruitment of cofilin was also significantly impaired in LY294002-treated slices (Fig. 2E, F, “cLTP” panel). These results indicate that PI3K catalytic activity is required for both basal and activity-dependent concentration of cofilin into spines.

To test the interaction of these mechanisms with the contribution of p85α, we combined the LY294002 treatment with co-transfection of the p85α shRNAs (shp85α (L + S) or shp85α (L)) and cofilin-GFP. As shown in Fig. 2E, F (“Baseline” panel), basal cofilin concentration in spines was significantly reduced in an additive manner to the individual treatments. That is, shp85α (L + S), which on its own reduced cofilin spine accumulation, was further reduced in the presence of LY294002 (compare pink versus grey symbols; values for shp85α and shp85β expression are also replotted here, for comparison). And shp85α (L), which on its own did not alter cofilin distribution, in the presence of LY294002 produced the same impairment as LY294002 alone (compare orange versus grey symbols). Similarly, cofilin recruitment after cLTP induction was further impaired with shp85α (L + S) or shp85α (L) plus PI3K inhibition, relative to the individual manipulations (Fig. 2E, F; “cLTP” panel; pink and orange symbols). Again, the effect was strongest with shp85α (L), which on its own significantly reduced cLTP-induced cofilin recruitment, and in the presence of LY294002 virtually abolished it (compare red and orange symbols). Therefore, these additive effects suggest that p85α and the catalytic activity of PI3K contribute to the activity-dependent recruitment of cofilin into spines through independent mechanisms.

Finally, we evaluated the effect of blocking PI3K activity on the enhancement of cLTP-induced cofilin recruitment into spines observed with shp85β. Indeed, the effect of shp85β in combination with LY294002 was intermediate between the separate treatments, that is, the accelerated cofilin recruitment in shp85β condition and the impairment produced by LY294002 (Fig. 2E, F, “cLTP” panel; magenta symbols).

Therefore, these combined findings suggest that p85 regulatory subunits contribute to the cofilin recruitment into spines during cLTP by separate (or additive) mechanisms to those carried out by the p110 catalytic subunit of PI3K. To note, none of these manipulations, including combinations of shRNAs and pharmacological inhibitors, altered spine density (Suppl. Figure 2B).

### p85α BH domain binds Rac1 and mediates cofilin recruitment into spines

Our results shown above indicate that p85α, and not p85β, is required for LTP and for cLTP-induced cofilin recruitment at spines. In addition, these data also suggest that this function probably resides in the N-terminal region of p85α, since the strongest effects were observed with shp85α (L) (in the presence of p55α and p50α, which share the C-terminal domains with p85α [47, 49]). Within this N-terminal region, the BH domain (BCR homology domain) is the one that presents the lowest percentage of homology between p85α and p85β [51]. This domain contains the Rac effector sequence [39]. Therefore, we directly tested whether the BH domains of p85α and p85β (hereinafter called BHα and BHβ, respectively) bind Rac1 and/or Cdc42, and whether there are differences between them.

**Fig. 3:**
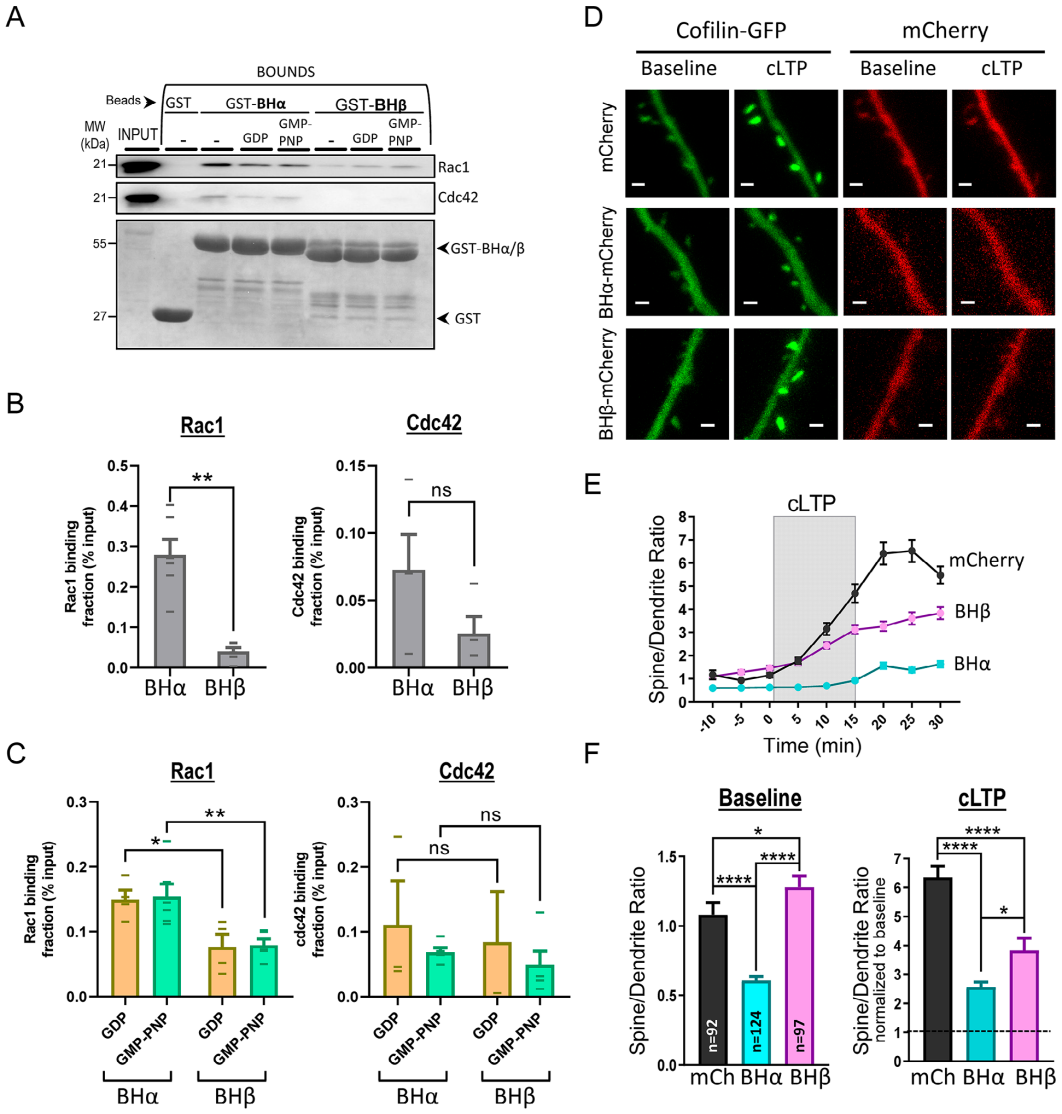
Interaction of the BH domains of p85α and p85β with Rac1 and Cdc42, and effect on GFP-cofilin recruitment into spines. **(A)** Hippocampal extracts were incubated with GST fusion proteins of the BH domain of p85α (BHα) or p85β (BHβ) as baits. GST alone was used as negative control. Some extracts were incubated with GDP or with the GTP analog GMP-PNP, as indicated. Bound and input fractions were analyzed by Western blot using specific antibodies for Rac1 and Cdc42. Lower blot shows GST protein expression. **(B)** Quantification of Rac1 (*left*) and Cdc42 (*right*) levels in the bound fraction, relative to the input protein, pulled-down with BHα or BHβ from extracts without nucleotide incubation. Individual values for each experiment are represented as lines overlying the columns. Bars represent mean ± SEM. **(C)** Similar to (B), with extracts incubated with GDP or GMP-PNP. **(D)** Representative images of dendritic branches from CA1 hippocampal neurons expressing GFP-cofilin (green channel) together with BHα or BHβ fused to mCherry (red channel). mCherry alone is used as control. Images are shown for baseline and after cLTP induction (15–30 min). Scale bar: 1 μm. **(E)** Quantification of GFP fluorescence signal in spines relative to the adjacent dendritic shaft (spine/dendrite ratio) before, during (grey rectangle) and after cLTP induction, from neurons expressing BHα (blue symbols), BHβ (pink symbols) or mCherry control (black symbols). Plot represents mean ± SEM. **(F)** Histogram plot of spine/dendrite ratios from baseline (*left*; -10–0 min) and after cLTP induction (*right*; 15–30 min, normalized to baseline values for each condition). Bars show mean ± SEM. n represents number of spines from 4–5 independent experiments in each condition. Statistical differences between conditions were evaluated through Kruskal-Wallis and Mann-Whitney U tests. ****p < 0.0001, *p < 0.05, ns: not significant

To this end, we performed pull-down assays from rat hippocampal extracts using GST fused to BHα or BHβ as baits. GST alone-containing beads were used as negative control for binding specificity. As shown in Fig. 3A, B, Rac1 was pulled-down much more efficiently with BHα, as compared to BHβ (a similar trend was observed for Cdc42, but the difference was not statistically significant, perhaps because of the overall weaker binding). We also tested whether this interaction was dependent on the activation state of the small GTPase. As shown in Fig. 3A, C, the extent of Rac1 and Cdc42 binding was equivalent with extracts preincubated with GDP (for the inactive form of the small GTPase) or with the GTP analog GMP-PNP (for its active form). Therefore, these results confirm that the BH domain of p85 does bind Rac1/Cdc42 and indicate that this interaction is stronger for p85α, as compared to p85β. Interestingly, this binding appears to be independent from the activity state of the GTPase (GDP- or GTP-bound), at least when assaying isolated BH domains (to note, specific binding for the GTP-bound forms has been reported previously using full-length p85 [39, 40]).

Given the preferential binding of Rac1 and Cdc42 to the BH domain of p85α, we hypothesized that this interaction may be involved in the recruitment of cofilin into spines. If this is the case, overexpression of the isolated BH domain may act as dominant negative by outcompeting the interactions with the endogenous p85α/β BH domains. To test this idea, organotypic hippocampal slices were cotransfected with cofilin-GFP together with BHα or BHβ domains fused to mCherry (or mCherry alone, as control), and we carried out live-cell imaging experiments during cLTP induction, as described before (see representative images in Fig. 3D). As shown in Fig. 3E, F (“Baseline” panel), cofilin was significantly less concentrated at spines under basal conditions when the BHα domain was overexpressed (blue symbols), whereas the BHβ domain produced a slight (but statistically significant) increase in cofilin spine concentration (pink symbols). After cLTP induction, both BH domains reduced cofilin recruitment, but the impairment was significantly stronger in the case of BHα overexpression (Fig. 3E, F, “cLTP” panel).

These results strongly suggest that the BH domain of p85 is an important factor for cofilin recruitment into spines. This function is carried out by p85α, in line with the preferred interaction of BHα with the small GTPases Rac1 and Cdc42.

### p85α is required for PI3K-induced Rac1 activation and actin polymerization in dendritic spines

PI3K catalytic activity (from the p110 subunit) has been shown to contribute to Rac1/Cdc42 activation via PI(3,4,5)P_3_-mediated regulation of specific guanine nucleotide exchange factors (GEFs), such as P-Rex, Vav, Tiam (reviewed in [52]). On the other hand, our results above suggest that p85α may provide additional mechanisms for activation, based on its direct interaction with these small GTPases. Therefore, we decided to test whether p85 subunits (and specifically p85α) has a net contribution to Rac1 activation during PI3K activation. To this end, we used hippocampal primary neuronal cultures infected with the different p85 shRNAs and treated with peroxovanadate, which produces a generalized activation of PI3K by blocking phospho-Tyr phosphatases [53]. Then, Rac1 activation was assessed by pull-down assays using the Rac1/Cdc42-binding domain of PAK1 fused to GST (GST-PAK-PBD beads; see Suppl. Figure 3A for the specificity of this assay to pull-down active Rac1/Cdc42).

**Fig. 4:**
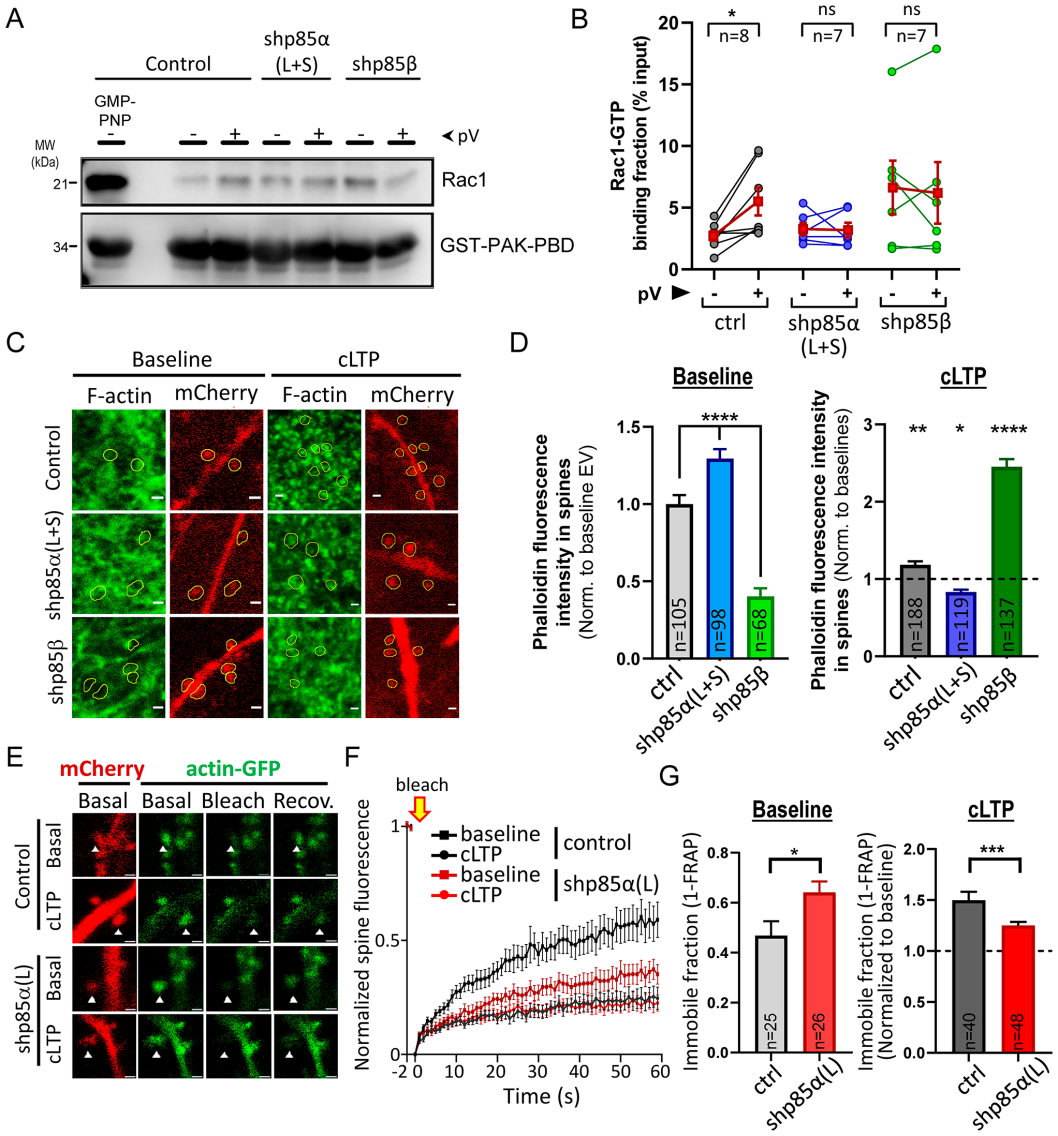
Effect of p85 shRNAs on Rac1 activation and F-actin accumulation and dynamics in spines. **(A)** Pull-down of active (GTP-bound) Rac1 with GST-PAK-PBD from extracts of hippocampal neurons treated or not with peroxovanadate (pV) and expressing shp85α (L + S), shp85β or an empty vector. Some untreated extracts were incubated with the GTP analog GMP-PNP, as control for active Rac1. Bound fractions were analyzed by Western blot using an antibody specific for Rac1. Lower blot shows GST protein expression. **(B)** Quantification of Rac1 levels in the bound fraction, relative to the input protein. Individual values for each experiment are represented as line-connected circles between untreated and peroxovanadate-treated conditions. Red squares represent mean ± SEM. n: number of independent experiments. Significant differences between conditions were assessed by Wilcoxon t-test. *p < 0.05, ns: not significant. **(C)** Representative images of organotypic hippocampal slices stained for F-actin with phalloidin-488 (green channel), expressing shp85α (L + S), shp85β or an empty vector coupled to mCherry expression (red channel). Images are shown for baseline (untreated) slices and for slices after cLTP induction (15–25 min). Dendritic spines, identified from the mCherry channel, are indicated with yellow shapes. Scale bar: 1 μm. **(D)** Histogram plot of phalloidin fluorescence intensity at individual spines from infected neurons detected from the mCherry signal, from baseline (*left*; -10–0 min) and after cLTP induction (*right*; 15–25 min, normalized to baseline values for each condition). Bars represent mean ± SEM. n represents number of spines from 8–12 different slices. Significant differences between conditions were assessed by Mann-Whitney U test. ****p < 0.0001, **p < 0.01, *p < 0.05. **(E)** Representative images of dendritic branches from CA1 hippocampal neurons expressing actin-GFP (green channel) together with shp85α (L) or the empty vector co-expressing mCherry (red channel). Images are shown at baseline (“Basal”), immediately after photobleaching (“Bleach”) and after 1 min of fluorescence recovery (“Recov.”), from both untreated slices or after 15 min of cLTP induction. Scale bar: 1 μm. Dendritic spines selected for photobleaching are indicated with arrowheads. **(F)** Time course of Fluorescence Recovery After Photobleaching (FRAP) at individual spines from control neurons or neurons expressing shp85α (L), before and after cLTP induction. Fluorescence intensity is normalized to baseline values and residual fluorescence immediately after photobleaching. Bars represent mean ± SEM. **(G)** Histogram plot of the fraction of immobile actin-GFP (1-FRAP, from the end of the time course) at individual spines from control neurons or from neurons expressing shp85α (L), before (“Baseline”) and after cLTP induction. Values for cLTP are normalized to their corresponding baseline. Bars represent mean ± SEM. n represents number of spines from 7 (empty vector control) or 9 (shp85α (L)) different slices. Significant differences between conditions were assessed by Mann-Whitney U test. **p < 0.01, *p < 0.05

Peroxovanadate treatment did produce a significant Rac1 activation (Fig. 4A, B; “control”), together with a strong activation of the PI3K pathway (monitored by Akt phosphorylation; Suppl. Figure 3B, C; “control”). In contrast, peroxovanadate-induced Rac1 activation was significantly blunted by p85α knock-down (Fig. 4A, B), despite the fact that PI3K catalytic activity was still present (Suppl. Figure 3B, C). Interestingly, shp85β also prevented Rac1 activation after peroxovanadate treatment (Fig. 4A, B). However, in this case, p85β knock-down appeared to produce a global activation of Rac1, although this effect was not statistically significant. Therefore, these results allow us to conclude that PI3K catalytic activity is insufficient to drive Rac1 activation in neurons in the absence of p85α.

Finally, we decided to test whether these specific effects of p85 isoforms on Rac1 activation and cofilin recruitment into spines had functional consequences for the regulation of the actin cytoskeleton. To test this idea, we used phalloidin staining in organotypic hippocampal slices to analyze the polymerization state of actin in dendritic spines of CA1 pyramidal neurons after cLTP induction, while reducing p85α or p85β levels. In this experiment, dendritic spines were selected from their mCherry fluorescence (co-expressed with the shRNAs; see representative images in Fig. 4C, red channel), and therefore, the analysis was effectively blind with respect to their phalloidin staining (Fig. 4C, green channel). As shown in Fig. 4D (“Baseline”), shp85α (L + S) produced a significant increase in F-actin in spines, whereas shp85β produced the opposite effect, as compared with control neurons. However, upon cLTP induction, the effects were completely reversed. As expected, in the control condition (empty vector), cLTP induction led to an increase in actin polymerization (F-actin staining) in spines (Fig. 4D; “cLTP”, grey symbols). This increase in F-actin was completely abolished with the shp85α (L + S) (in fact, a significant reduction in F-actin staining was observed upon cLTP induction), whereas it was greatly enhanced with the shp85β (Fig. 4D; “cLTP”, blue and green symbols). These results parallel those observed for cofilin recruitment, and strongly suggest that actin polymerization in spines during cLTP is driven preferentially by p85α.

As a complementary approach to evaluate the role of p85α in actin dynamics during LTP, we carried out live imaging of actin-GFP in dendritic spines and used FRAP (Fluorescence Recovery After Photobleaching) to assess the fraction of mobile and immobile actin before and after cLTP. Organotypic hippocampal slices were cotransfected with actin-GFP and with shp85α (L) to specifically knock-down the p85α long isoform, or with the empty vector as control. mCherry expression from the shRNA and control plasmids served to confirm cotransfection (see representative image in Fig. 4E). Individual spines expressing actin-GFP were photobleached before (baseline) or 15 min after cLTP induction, and the extent of fluorescence recovery was monitored. Under basal conditions, approximately 50% of actin-GFP fluorescence signal was recovered after 60 s post-bleaching (Fig. 4F, G, “Baseline”), indicating that over this period of time, approximately half of actin-GFP is stable in dendritic spines. The fraction of immobile actin became significantly larger after cLTP induction (Fig. 4F, G, “cLTP”), probably reflecting an increase in stable actin filaments in spines after LTP. Importantly, this LTP-dependent actin stabilization was significantly impaired in neurons expressing shp85α (L), consistent with the failure to enhance F-actin labeling after cLTP in the absence of p85α (Fig. 4D).

Therefore, these combined data indicate that actin polymerization and F-actin stabilization in dendritic spines during LTP are strongly dependent on p85α.

## Discussion

In this work, using a knockdown strategy on CA1 hippocampal neurons, we have found that the p85α regulatory isoform of PI3K is required for LTP, but not for mGluR LTD. We propose that the role of p85α in LTP is linked to its contribution to structural plasticity. Thus, we have found that PI3K activity triggers Rac1 activation in neurons only when associated to p85α. In agreement with this observation, p85α is required for cofilin recruitment and F-actin polymerization in spines induced by cLTP. Importantly, these actions are specific for p85α and are not shared by the shorter splicing isoforms p55α/p50α (encoded by the same gene, *PIK3R1*) or by the paralog p85β (encoded by a different gene, *PIK3R2*).

The specificity for p85α is perhaps most strikingly demonstrated by the fact that removing p85β from hippocampal neurons produced not null, but opposite effects from those observed upon p85α depletion. Thus, shp85β led to accelerated cofilin recruitment and stronger F-actin polymerization in spines upon cLTP induction, together with faster synaptic potentiation. We believe these enhancing effects of the shp85β reflect the intrinsic partition of PI3K heterodimers between those containing p85α and p85β. This is because we have observed that removal of p85β does not reduce the levels or the heterodimerization extent of the catalytic p110 subunits, but shifts their association towards p85α. Therefore, this manipulation will act as a gain-of-function change, by increasing the amount of p110/p85α complexes, which would be the ones mediating the synaptic effects of p85. Importantly, this interpretation also implies that the availability of endogenous p85α-containing PI3K complexes is a limiting factor for synaptic potentiation and structural plasticity during LTP.

**Fig. 5:**
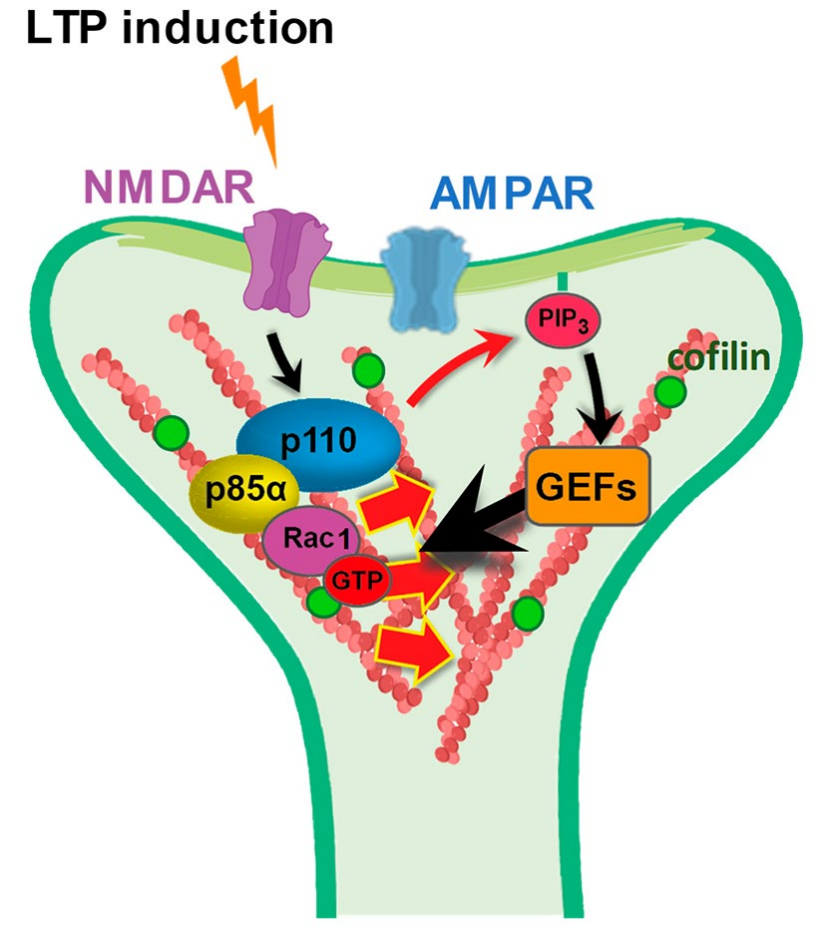
Cartoon model for p85α-driven Rac1 action at dendritic spines. Specific association of Rac1 with the PI3K regulatory subunit p85α favors local remodeling of the actin cytoskeleton at the synaptic compartments where PI3K catalytic activity is engaged

What is the specific contribution of p85α to these mechanisms? Some differing functions of p85α and p85β have been described before, mostly related to Akt signaling during cell proliferation [54–58]. These differences have often been attributed to small sequence variations in the C-terminal SH_2_ domains of p85α and p85β, which regulate p110 catalytic activity [54, 55]. In this study, however, we are proposing a p85-specific mechanism based on the preferential interaction we have observed between the small GTPases Rac1/Cdc42 and the N-terminal BH domain of p85α. To note, the interaction between p85 and Rac1/Cdc42 had been described before [39, 40], but surprisingly, the BH domains of p85α and p85β had never been directly compared for their Rac1/Cdc42 binding, despite the fact that the BH domain contains the most divergent sequences between p85α and p85β. Once established the preferential binding of Rac1 to p85α, we have observed that this interaction is functionally relevant, as p85β depletion (favoring p110/p85α association) triggers a much stronger F-actin polymerization in spines upon cLTP induction. How could this PI3K-Rac1/Cdc42 interaction favor the action of these small GTPases at synapses? We believe that, in this context, p85 is acting as a scaffold protein controlling the localization of Rac1/Cdc42 and subsequent actin remodeling at the precise locus where PI(3,4,5)P_3_ synthesis occurs. Thus, when PI3K catalytic activity is induced during LTP [30, 59], activation of PI(3,4,5)P_3_-sensitive Rac1 GEFs (such as Tiam1 [60, 61]) will focus Rac1 actions at the synaptic compartments where PI3K activation is taking place (see model in Fig. 5). On the other hand, this process may synergize with additional mechanisms independent from PI(3,4,5)P_3_, since we have also observed p85α-dependent cofilin recruitment in spines while blocking PI3K catalytic activity. Importantly, these mechanisms can only be supported by the long p85 isoforms, as they contain a BH domain. This interpretation may also explain why depletion of p85α together with p55α/p50α (shp85α (L + S)) had a milder phenotype than removing exclusively p85α (shp85α (L)). In the combined absence of p85α, p55α and p50α isoforms, p110 would only form complexes with p85β, whose BH domain may also interact to some extent with Rac1/Cdc42, even if less efficiently than p85α. In contrast, the presence of p50α/p55α would be detrimental for LTP and structural plasticity, because these short isoforms generate PI3K complexes deprived from Rac1-assisted mechanisms. In this sense, p50α/p55α subunits act as dominant negative forms for Rac1-dependent functions of the PI3K complex.

And what would be the connection of these mechanisms with cofilin recruitment into spines during cLTP? It is really not known what triggers the translocation of cofilin into dendritic spines upon LTP induction. However, it does seem that its retention within spines is determined by its phosphorylation at Ser3 [23], which in turn inhibits cofilin severing activity against actin filaments [62]. Therefore, in its inactive (phosphorylated) form, cofilin binding to F-actin contributes to actin filament stabilization [24] and results in cofilin retention in the activated spines [21]. Cofilin phosphorylation is catalyzed by LIM kinase, which is in turn activated by Rac1 and Cdc42 [20, 42, 63]. In this scenario, the action of p85α facilitating Rac1 activation by PI3K will overall contribute to cofilin retention and F-actin stabilization in potentiated spines.

In conclusion, this study has provided mechanistic insight into additional functionalities specifically contributed by the regulatory subunit p85α for PI3K signaling during synaptic plasticity. Our results also highlight how the balance between p85α- and p85β-containing PI3K complexes may determine the direction of this signaling. Similarly, the relative expression of the long (p85α) and the short (p55α/p50α) splicing isoforms of the *PIK3R1* gene may also alter the contribution of Rac1 to PI3K signaling. These considerations open the intriguing possibilities that neuronal function and cognitive performance may be altered if the relative expression of p85α and p85β changes (as it has been observed under some pathological conditions [64, 65]) or if *PIK3R1* alternative splicing is modified (as it has been reported for some genetic disorders [66]).

## Methods

### Organotypic hippocampal slice cultures

Tissue slices were prepared from 5-7-day-old Wistar rat pups essentially as described previously [67, 68]. Briefly, whole brains were removed in ice-cold dissection medium (10 mM D-glucose, 4 mM KCl, 20 mM NaHCO_3_, 234 mM sucrose, 5 mM MgCl_2_, 1 mM CaCl_2_) previously gassed with carbogen (5% CO_2_/95% O_2_). After removal of the hippocampi, 400 μm-thick slices were prepared using a tissue slicer (Stoelting Europe) under sterile conditions. Individual slices were transferred to porous nitrocellulose permeable membranes (Merk Millipore) over slice culture medium (0.8% [wt/vol] MEM powder, 20% [vol/vol] horse serum, 1 mM L-glutamine, 1 mM CaCl_2_, 2 mM MgSO_4_, 1 mg/L insulin, 0.0012% [vol/vol] ascorbic acid, 30 mM Hepes, 13 mM D-glucose, and 5.2 mM NaHCO_3_)). Slices were maintained in vitro at 35.5 ºC and 5% CO_2_ for 7–15 days until use and culture medium was refreshed every 2–3 days.

### Primary hippocampal neuronal cultures

Dissociated cultures were prepared from E18-E19 rat embryos essentially as described previously [69]. Briefly, hippocampi were removed in Hank’s balanced salt solution (Gibco) supplemented with 10 mM HEPES pH 7.4 (Gibco), 100 IU/mL penicillin and 100 µg/mL streptomycin. Cells were disaggregated by trypsinization with 0.25% trypsin (Gibco) and mechanically dispersed by repeated passage through pipette tips of different thickness. Neurons were then counted and plated at a density of 21,000–28,000 cells/cm^2^ on 0.1 mg/mL poly-L-lysine-coated culture plates for biochemical analysis. The attachment to the substrate and recovery of cells were performed in MEM containing 1.5% D-glucose and 10% FBS (Hyclone) for 3–4 h at 37 ºC and 5% CO_2_. The media was then replaced with Neurobasal medium (Gibco) supplemented with 1x B27 (Gibco) and 2 mM glutamine. Cultures were maintained at 37 °C and 5% CO_2_ and used after 2 weeks in vitro.

### Antibodies and other reagents

For western blot, the following antibodies were used: p110β (#ab151549), p85α (#ab191606) and p85β (#ab180967) purchased from Abcam. Antibodies against p110α (#4255), phospho-Akt [Thr308] (#2965), phospho-Akt [Ser473] (#4060) and pan Akt (#2910) were purchased from Cell Signalling Technologies. Antibodies raised against p85 (#ABS234), actin (#MAB1501R) and GFP (#11814460001) were purchased from Merck Millipore. Antibodies to Rac1 (#610651) and Cdc42 (#610928) were acquired from BD Biosciences. Other antibodies used for immunodetection were anti-mCherry (GeneTex, #GTX59788) and anti-GST (Sigma-Aldrich, #G7781). Secondary antibodies used for western blot were horseradish peroxidase (HRP)-conjugated anti-rabbit (#711-035-152) and anti-mouse (#715-035-151), purchased from Jackson ImmunoResearch.

LY294002 (#L9908), forskolin (#F6886), picrotoxin (#P1675), rolipram (#R6520), DL-AP5 (#A5282) and 2-chloroadenosine (#C5134) were purchased from Sigma/Merck-Millipore.

### DNA constructs

#### shRNA constructs: shp85α (L + S), shp85α (L) and shp85β

For shRNA knockdown of the different rat p85 isoforms (p85α, p55α, p50α and p85β), the lentiviral vector KH1-LV-mCherry vector, gift from Dr. María S. Soengas (Centro Nacional de Investigaciones Oncolológicas, CNIO-ISCIII, Madrid), was used. The specific shRNA targets were: shp85α (L + S), 5’-GCATGAACAACAATATGTCCT-3’; shp85α (L), 5’-GGCACTTGGATTCAGTGATGG-3’; shp85β, 5’-GCGGGAACAACAAGCTAATCA-3’. As negative control, the KH1-LV-mCherry vector with no target sequence (empty vector) was used.

#### Recombinant proteins

The cofilin-GFP plasmid contains the full-length sequence for human cofilin fused to the GFP protein. It is controlled by the CMV promoter and it was generously provided by Dr. Petronila Penela (Centro de Biología Molecular Severo Ochoa, CSIC-UAM, Madrid). p85 BH domains were isolated by PCR from the corresponding full-length p85α and p85β cDNA sequences (residues R79 to W333 for BHα -NCBI Reference Sequence: XP_016865074.1; residues P80 to S324 for BHβ - NCBI Reference Sequence: NP_005018.2). PCR products were cloned into the pmCherry-C1 vector (to generate the mCherry-BHα and mCherry-BHβ plasmids) or into the pGEX-2T vector (to generate the GST-BHα and GST-BHβ plasmids).

### Pharmacological treatments

#### Induction of NMDAR-depending chemical LTP (cLTP)

cLTP (forskolin-induced LTP) was carried out as previously described [50, 70]. Briefly, organotypic hippocampal slices were transferred to a submersion-type holding chamber containing ACSF (119 mM NaCl, 2.5 mM KCl, 4 mM CaCl_2_, 4 mM MgCl_2_, 26 mM NaHCO_3_, 1 mM NaH_2_PO_4_, 11 mM glucose, pH 7.4 and osmolarity adjusted to 290 ± 5 mOsm) gassed with carbogen (5% CO_2_/95% O_2_) at 29ºC. For cLTP induction, slices were transferred to a Mg^2+^-free ACSF supplemented with rolipram (0.1 µM), forskolin (50 µM) and picrotoxin (100 µM) during 15 min. For some experiments, hippocampal slices were returned back to regular ACFS for a recovery period (or wash period) of 10–15 min after induction. In the case of dissociated hippocampal neurons, HEPES buffer was used in the ACSF to avoid continuous carbogen gassing.

#### Induction of PI3K activity in primary cultures

After 1 h incubation in medium without serum (to decrease basal activation of receptor tyrosine kinases), primary hippocampal cultures were treated with peroxovanadate (mix of 13.2 mM Na_3_VO_4_, 13.2 mM H_2_O_2_, 40 mM HEPES) for 10 min at 37 ºC in Tyrode’s solution (30 mM glucose, 120 mM NaCl, 5 mM KCl, 2 mM MgCl_2_, 2 mM CaCl_2_, 25 mM HEPES) (1 µL peroxovanadate solution: 240 µL Tyrode’s solution ratio).

### GST pull-down assays

Primary hippocampal neurons (DIV12-14) were lysed in lysis buffer (20 mM Tris-HCl pH 7.5, 150 mM NaCl, 1% Triton X-100, 1 mM DTT, 2x protease inhibitor cocktail (Complete Tablets EDTA-free, EASYpack, Roche), 1x phosphatase inhibitor cocktail (PhosSTOP EASYpack, Roche), 10 mM MgCl_2_). Protein extracts were prepared at 0.1–0.5 µg/µL (200–500 µL). For nucleotide controls, some lysates were incubated with 10 mM EDTA (40 min at room temperature) followed by addition of 1 mM GDP (SIGMA) or 0.1 mM GMP-PNP (guanosine-5’-[β,γ-imido]triphosphate, Jena Bioscience) in the presence of excess (60 mM) MgCl_2_ (1 h on ice). Glutathione beads bound to the corresponding GST-fusion protein were incubated with 50 µg of the different lysates for 1 h at 4 ºC with rotation. GSH-beads with GST alone were used as a negative control. Then, beads with the bound proteins were pelleted by centrifugation (2400 rpm for 2 min at 4 ºC), washed and resuspended in 20 µL cold lysis buffer (bound fraction). Protein extracts were finally subjected to 12% SDS-PAGE and analyzed by Western Blot.

### p110α co-immunoprecipitation assays

Organotypic hippocampal slices or dissociated hippocampal neurons were homogenized in cold lysis buffer (20 mM Tris-HCl pH 7.5, 150 mM NaCl, 1% Triton X-100, 1 mM EGTA, 1 mM EDTA, 5 mM MgCl_2_, 2x protease inhibitors cocktails (Complete Tablets EDTA-free, EASYpack, Roche) and 1x phosphatase inhibitor cocktail (PhosSTOP EASYpack, Roche) and protein extracts were prepared at 0.5 µg/µL (400–500 µL). Extracts were incubated with anti-p110α antibody (119 µg/mL, Cell Signaling 4255 S) and 50 µL of Protein G Sepharose beads (GE Healthcare) equilibrated at 50% in homogenization buffer. Bound fractions were isolated by centrifugation (2400 rpm for 2 min) followed by three washes and resuspension in 20 µL lysis buffer. All fractions were then separated by SDS–PAGE and analyzed by Western blot.

### Western blotting

Protein extracts were processed by SDS–polyacrylamide gel electrophoresis and transferred to polyvinylidene fluoride (PVDF) membranes (Immobilon-P, Merck Millipore). Afterwards, membranes were blocked (5% w/v non-fat dry milk in tris-buffered saline (TBS) + 0.1% Tween-20) (1 h at room temperature) and incubated with primary antibodies overnight at 4 ºC. After a 45-minutes incubation with horseradish peroxidase (HRP)-conjugated secondary antibodies, immunodetection was done by chemiluminescence with 5-minutes ECL incubation (Enhanced ChemiLuminiscence, Immobilon Western, Millipore) and the ImageQuant™ LAS 4000 mini biomolecular imager (GE Healthcare Life Sciences). The ImageJ software was used to analyze and quantify signal intensities obtained from digital images.

### Electrophysiology

Excitatory postsynaptic currents (EPSCs) were recorded from CA1 pyramidal neurons with glass recording electrodes while stimulating Schaffer collateral fibers using single-voltage pulses (200 µs, up to 25 V). During the recordings, the slices were placed in an immersion chamber constantly perfused with ACSF gassed with 5% CO_2_ and 95% O_2_ at 29 °C. For all experiments, ACSF was supplemented with 100 µM picrotoxin and 4 µM 2-chloroadenosine. Recordings were carried out under whole-cell voltage-clamp configuration using glass micropipettes. These patch recording pipettes (3–6 MΩ) were filled with an internal solution containing 115 mM CsMeSO_3_, 20 mM CsCl, 10 mM Hepes, 2.5 mM MgCl_2_, 4 mM Na_2_-ATP, 0.4 mM Na-GTP, 10 mM sodium phosphocreatine, 0.6 mM EGTA, and 10mM lidocaine N-ethyl bromide, pH 7.25, osmolarity 290 mOsm. For paired recordings, infected and uninfected (control) neurons were identified through fluorescence illumination and then patched and recorded simultaneously. Only CA1 neurons were infected with lentiviruses, thus ensuring that shRNAs were expressed exclusively in the postsynaptic cell when measuring CA3 to CA1 synaptic transmission. Synaptic AMPAR-mediated responses were measured as the peak amplitude of the response at -60 mV. NMDAR-mediated responses were recorded at + 40 mV and measuring the tail response at a point when AMPAR-mediated responses had fully decayed (65 ms post-stimulation). Synaptic responses were averaged over 50–70 trials. LTP was induced by pairing presynaptic stimulation (300 pulses at 3 Hz) with a depolarization of the postsynaptic cell to 0 mV [71]. mGluR-dependent LTD was induced in presence of 100 µM DL-AP5 with a paired-pulse protocol [72] (900 pairs of pulses, with a 50 ms inter-stimulus interval, at 1 Hz). Data acquisition was carried out with MultiClamp 700 A/B amplifiers and pClamp software (Molecular Devices). Data analysis was performed using custom-made Excel (Microsoft) macros. Series and input resistance of the recorded cells were monitored throughout the recording. Cells with changes of > 20% on series resistance, artifact change or burst firing events were discarded from the quantification.

### Live cell imaging assays and quantification

Fluorescence images were acquired focusing mainly on dendritic spines located in secondary to quaternary dendritic segments of CA1 hippocampal pyramidal neurons. Biolistically-cotransfected organotypic hippocampal slices were placed in a chamber continuously perfused with ACSF gassed with carbogen (5% CO_2_/95% O_2_) at 29 °C. Confocal fluorescence images were acquired as Z-stacks with a confocal inverted microscope (LSM 800, Zeiss) using a 63x NA 1.2 water C-Apochromat objective, a 2x zoom factor and 488-nm and 561-nm lasers in combination with ZenBlue 3.2 software. For Fluorescence Recovery After Photobleaching (FRAP) experiments, individual spines were bleached for 2 s at maximal laser intensity. Recovery of fluorescence in the spine was monitored up to 60 s after photobleaching. This process was repeated on several spines from different neurons under baseline conditions and after 15 min of cLTP induction.

For image analysis and quantification, Z-stacks were reconstructed (maximum intensity projection) using the software Fiji v1.52n (ImageJ; National Institutes of Health). Spine/dendrite ratios of GFP signals were calculated after background signal subtraction. For spine density analysis, spines were counted via a cell counter ImageJ plug-in whereas dendritic segments were traced and measured via the ImageJ segmented line tool. Finally, spine density was obtained by dividing the number of spines by the length of the dendritic segment.

### Phalloidin labelling

Hippocampal slices were fixed with 4% paraformaldehyde (PFA) and 4% sucrose in PBS pH 7.4 for 1 h at room temperature. Subsequent steps were performed with washes with PBS between them. Fixed samples were then blocked (3% BSA, 3% horse serum and 0.1% Triton X-100) for 1 h at room temperature followed by incubation with 132 nM (1:100) Alexa Fluor 488-conjugated phalloidin (ThermoFisher) in PBS overnight at 4 ºC. After final washes (3 × 10 min), samples were mounted onto microscope slides and fluorescence images were acquired as 5 μm-depth Z-stacks starting at the same level from the surface among the different slices. All images were acquired using the same microscope settings and conditions. A confocal microscope (LSM 800, Zeiss) was used with a 63x NA 1.2 water C-Apochromat objective, a 2x zoom factor and 488-nm and 561-nm lasers in combination with ZenBlue 3.2 software. As with live-cell imaging data, Z-stacks were reconstructed (maximum intensity projection) and analysed using the Fiji v1.52n software.

### Statistical analysis and figure representation

Statistical differences (*p*-values) between groups were determined via different non-parametric tests: Mann-Whitney U test for unpaired data, Wilcoxon test for normalised paired data and ANOVA/Kruskal-Wallis for comparing multiple groups. All statistical analysis and graphic representations were performed using GraphPad Prism software.

### Electronic supplementary material

Below is the link to the electronic supplementary material.

Supplementary Material 1

## Data Availability

All original data supporting the results reported in the article are available upon request.

## References

[1] Citri A, Malenka RC (2008) Synaptic plasticity: multiple forms, functions, and mechanisms. Neuropsychopharmacol off Publ Am Coll Neuropsychopharmacol 33:18–41. 10.1038/sj.npp.1301559 10.1038/sj.npp.1301559 17728696

[2] Bliss TVP, Collingridge GL (1993) A synaptic model of memory: long-term potentiation in the hippocampus. Nature 361:31–39. 10.1038/361031a0 8421494 10.1038/361031a0

[3] Martin SJ, Grimwood PD, Morris RGM (2000) Synaptic plasticity and memory: an evaluation of the hypothesis. Annu Rev Neurosci 23:649–711. 10.1146/annurev.neuro.23.1.649 10845078 10.1146/annurev.neuro.23.1.649

[4] Yang Y, Liu J-J (2022) Structural LTP: Signal transduction, actin cytoskeleton reorganization, and membrane remodeling of dendritic spines. Curr Opin Neurobiol 74:102534. 10.1016/j.conb.2022.102534 35398661 10.1016/j.conb.2022.102534

[5] Bosch M, Hayashi Y (2012) Structural plasticity of dendritic spines. Curr Opin Neurobiol 22:383–388. 10.1016/j.conb.2011.09.002 21963169 10.1016/j.conb.2011.09.002 PMC4281347

[6] Penzes P, Cahill ME, Jones KA, Srivastava DP (2008) Convergent CaMK and RacGEF signals control dendritic structure and function. Trends Cell Biol 18:405–413. 10.1016/j.tcb.2008.07.002 18701290 10.1016/j.tcb.2008.07.002

[7] Holtmaat A, Svoboda K (2009) Experience-dependent structural synaptic plasticity in the mammalian brain. Nat Rev Neurosci 10:647–658. 10.1038/nrn2699 19693029 10.1038/nrn2699

[8] Bredt D, Nicoll RA (2003) AMPA receptor trafficking at excitatory synapses. Neuron 40:361–379. 10.1016/S0896-6273(03)00640-8 14556714 10.1016/S0896-6273(03)00640-8

[9] Malinow R, Malenka RC (2002) AMPA receptor trafficking and synaptic plasticity. Annu Rev Neurosci 25:103–126. 10.1146/annurev.neuro.25.112701.142758 12052905 10.1146/annurev.neuro.25.112701.142758

[10] Shepherd JD, Huganir RL (2007) The cell biology of synaptic plasticity: AMPA receptor trafficking. Annu Rev Cell Dev Biol 23:613–643. 10.1146/annurev.cellbio.23.090506.123516 17506699 10.1146/annurev.cellbio.23.090506.123516

[11] Snyder EM, Philpot BD, Huber KM et al (2001) Internalization of ionotropic glutamate receptors in response to mGluR activation. Nat Neurosci 4:1079–1085. 10.1038/nn746 11687813 10.1038/nn746

[12] Diering GH, Huganir RL (2018) The AMPA receptor code of synaptic plasticity. Neuron 100:314–329. 10.1016/j.neuron.2018.10.018 30359599 10.1016/j.neuron.2018.10.018 PMC6214363

[13] Carroll RC, Beattie EC, von Zastrow M, Malenka RC (2001) Role of AMPA receptor endocytosis in synaptic plasticity. Nat Rev Neurosci 2:315–324. 10.1038/35072500 11331915 10.1038/35072500

[14] Fukazawa Y, Saitoh Y, Ozawa F et al (2003) Hippocampal LTP is accompanied by enhanced F-actin content within the dendritic spine that is essential for late LTP maintenance in vivo. Neuron 38:447–460. 10.1016/S0896-6273(03)00206-X 12741991 10.1016/S0896-6273(03)00206-X

[15] Okamoto K-I, Nagai T, Miyawaki A, Hayashi Y (2004) Rapid and persistent modulation of actin dynamics regulates postsynaptic reorganization underlying bidirectional plasticity. Nat Neurosci 7:1104–1112. 10.1038/nn1311 15361876 10.1038/nn1311

[16] Harris KM (2020) Structural LTP: from synaptogenesis to regulated synapse enlargement and clustering. Curr Opin Neurobiol 63:189–197. 10.1016/j.conb.2020.04.009 32659458 10.1016/j.conb.2020.04.009 PMC7484443

[17] Nägerl UV, Eberhorn N, Cambridge SB, Bonhoeffer T (2004) Bidirectional activity-dependent morphological plasticity in hippocampal neurons. Neuron 44:759–767. 10.1016/j.neuron.2004.11.016 15572108 10.1016/j.neuron.2004.11.016

[18] Zhou Q, Homma KJ, Poo MM (2004) Shrinkage of dendritic spines associated with long-term depression of hippocampal synapses. Neuron 44:749–757. 10.1016/j.neuron.2004.11.011 15572107 10.1016/j.neuron.2004.11.011

[19] Stein IS, Zito K (2019) Dendritic spine elimination: Molecular mechanisms and implications. Neurosci Rev J Bringing Neurobiol Neurol Psychiatry 25:27–47. 10.1177/1073858418769644 10.1177/1073858418769644 PMC6167191 29716431

[20] Bernstein BW, Bamburg JR (2010) ADF/cofilin: a functional node in cell biology. Trends Cell Biol 20:187–195. 10.1016/j.tcb.2010.01.001 20133134 10.1016/j.tcb.2010.01.001 PMC2849908

[21] Bosch M, Castro J, Saneyoshi T et al (2014) Structural and molecular remodeling of dendritic spine substructures during long-term potentiation. Neuron 82:444–459. 10.1016/j.neuron.2014.03.021 24742465 10.1016/j.neuron.2014.03.021 PMC4281348

[22] Chen LY, Rex CS, Casale MS et al (2007) Changes in synaptic morphology accompany actin signaling during LTP. J Neurosci 27:5363–5372. 10.1523/JNEUROSCI.0164-07.2007 17507558 10.1523/JNEUROSCI.0164-07.2007 PMC6672340

[23] Noguchi J, Hayama T, Watanabe S et al (2016) State-dependent diffusion of actin-depolymerizing factor/cofilin underlies the enlargement and shrinkage of dendritic spines. Sci Rep 6. 10.1038/srep32897 10.1038/srep32897 PMC5011767 27595610

[24] Andrianantoandro E, Pollard TD (2006) Mechanism of actin filament turnover by severing and nucleation at different concentrations of ADF/cofilin. Mol Cell 24:13–23. 10.1016/j.molcel.2006.08.006 17018289 10.1016/j.molcel.2006.08.006

[25] Bamburg JR, Minamide LS, Wiggan O et al (2021) Cofilin and actin dynamics: multiple modes of regulation and their impacts in neuronal development and degeneration. 10.3390/cells10102726. Cells 10:10.3390/cells10102726 PMC8534876 34685706

[26] Pavlov D, Muhlrad A, Cooper J et al (2007) Actin filament severing by cofilin. J Mol Biol 365:1350–1358. 10.1016/j.jmb.2006.10.102 17134718 10.1016/j.jmb.2006.10.102 PMC2572264

[27] Cantley LC (2002) The phosphoinositide 3-kinase pathway. Sci (80-) 296:1655–1657 10.1126/science.296.5573.1655 12040186

[28] Arendt KL, Royo M, Fernández-Monreal M et al (2010) PIP3 controls synaptic function by maintaining AMPA receptor clustering at the postsynaptic membrane. Nat Neurosci 13:36–44. 10.1038/nn.2462 20010819 10.1038/nn.2462 PMC2810846

[29] Hou L, Klann E (2004) Activation of the phosphoinositide 3-kinase-akt-mammalian target of rapamycin signaling pathway is required for metabotropic glutamate receptor-dependent long-term depression. J Neurosci 24:6352–6361. 10.1523/JNEUROSCI.0995-04.2004 15254091 10.1523/JNEUROSCI.0995-04.2004 PMC6729543

[30] Man HY, Wang Q, Lu WY et al (2003) Activation of PI3-kinase is required for AMPA receptor insertion during LTP of mEPSCs in cultured hippocampal neurons. Neuron 38:611–624. 10.1016/S0896-6273(03)00228-9 12765612 10.1016/S0896-6273(03)00228-9

[31] Opazo P, Watabe AM, Grant SGN, O’Dell TJ (2003) Phosphatidylinositol 3-kinase regulates the induction of long-term potentiation through extracellular signal-related kinase-independent mechanisms. J Neurosci 23:3679–3688. 10.1523/jneurosci.23-09-03679.2003 12736339 10.1523/jneurosci.23-09-03679.2003 PMC6742185

[32] Sanna PP, Cammalleri M, Berton F et al (2002) Phosphatidylinositol 3-Kinase is required for the expression but not for the induction or the maintenance of long-term potentiation in the hippocampal CA1 region 10.1523/JNEUROSCI.22-09-03359.2002 PMC6758361 11978812

[33] Kim JI, Lee HR, Sim SE et al (2011) PI3Kg is required for NMDA receptor - dependent long-term depression and behavioral flexibility. Nat Neurosci 14:1447–1454. 10.1038/nn.2937 22019731 10.1038/nn.2937

[34] Peineau S, Taghibiglou C, Bradley C et al (2007) LTP inhibits LTD in the hippocampus via regulation of GSK3beta. Neuron 53:703–717. 10.1016/j.neuron.2007.01.029 10.1016/j.neuron.2007.01.029 17329210

[35] Sánchez-Castillo C, Cuartero MI, Fernández-Rodrigo A et al (2022) Functional specialization of different PI3K isoforms for the control of neuronal architecture, synaptic plasticity, and cognition. Sci Adv 8:eabq8109. 10.1126/sciadv.abq8109 36417513 10.1126/sciadv.abq8109 PMC9683729

[36] Enriquez-Barreto L, Cuesto G, Dominguez-Iturza N et al (2014) Learning improvement after PI3K activation correlates with de novo formation of functional small spines. Front Mol Neurosci 6. 10.3389/fnmol.2013.00054 10.3389/fnmol.2013.00054 PMC3877779 24427113

[37] Kumar V, Zhang MX, Swank MW et al (2005) Regulation of dendritic morphogenesis by Ras-PI3K-Akt-mTOR and Ras-MAPK signaling pathways. J Neurosci 25:11288–11299. 10.1523/JNEUROSCI.2284-05.2005 16339024 10.1523/JNEUROSCI.2284-05.2005 PMC6725910

[38] Lee C-C, Huang C-C, Hsu K-S (2011) Insulin promotes dendritic spine and synapse formation by the PI3K/Akt/mTOR and Rac1 signaling pathways. Neuropharmacology 61:867–879. 10.1016/j.neuropharm.2011.06.003 21683721 10.1016/j.neuropharm.2011.06.003

[39] Bokoch GM, Vlahos CJ, Wang Y et al (1996) Rac GTPase interacts specifically with phosphatidylinositol 3-kinase. Biochem J 315 (Pt 3:775–779. 10.1042/bj3150775 10.1042/bj3150775 PMC1217274 8645157

[40] Zheng Y, Bagrodia S, Cerione RA (1994) Activation of phosphoinositide 3-kinase activity by Cdc42Hs binding to p85. J Biol Chem 269:18727–18730 8034624 10.1016/S0021-9258(17)32226-3

[41] Fox M, Mott HR, Owen D (2020) Class IA PI3K regulatory subunits: p110-independent roles and structures. Biochem Soc Trans 48:1397–1417. 10.1042/BST20190845 32677674 10.1042/BST20190845 PMC7458397

[42] Yang N, Higuchi O, Ohashi K et al (1998) Cofilin phosphorylation by LIM-kinase 1 and its role in rac-mediated actin reorganization. Nature 393:809–812. 10.1038/31735 9655398 10.1038/31735

[43] Meng Y, Zhang Y, Tregoubov V et al (2002) Abnormal spine morphology and enhanced LTP in LIMK-1 knockout mice. Neuron 35:121–133. 10.1016/s0896-6273(02)00758-4 12123613 10.1016/s0896-6273(02)00758-4

[44] Rust MB (2015) ADF/cofilin: a crucial regulator of synapse physiology and behavior. Cell Mol Life Sci 72:3521–3529. 10.1007/s00018-015-1941-z 26037722 10.1007/s00018-015-1941-z PMC11113150

[45] Tohda C, Nakanishi R, Kadowaki M (2009) Hyperactivity, memory deficit and anxiety-related behaviors in mice lacking the p85alpha subunit of phosphoinositide-3 kinase. Brain Dev 31:69–74. 10.1016/j.braindev.2008.04.006 18538520 10.1016/j.braindev.2008.04.006

[46] Tohda C, Nakanishi R, Kadowaki M (2007) Learning deficits and agenesis of synapses and myelinated axons in phosphoinositide-3 kinase-deficient mice. Neurosignals 15:293–306. 10.1159/000108936 10.1159/000108936 17901711

[47] Fruman DA, Cantley LC, Carpenter CL (1996) Structural organization and alternative splicing of the murine phosphoinositide 3-kinase p85 alpha gene. Genomics 37:113–121. 10.1006/geno.1996.0527 8921377 10.1006/geno.1996.0527

[48] Antonetti DA, Algenstaedt P, Kahn CR (1996) Insulin receptor substrate 1 binds two novel splice variants of the regulatory subunit of phosphatidylinositol 3-kinase in muscle and brain. Mol Cell Biol 16:2195–2203. 10.1128/MCB.16.5.2195 8628286 10.1128/MCB.16.5.2195 PMC231207

[49] Inukai K, Funaki M, Ogihara T et al (1997) p85alpha gene generates three isoforms of regulatory subunit for phosphatidylinositol 3-kinase (PI 3-Kinase), p50alpha, p55alpha, and p85alpha, with different PI 3-kinase activity elevating responses to insulin. J Biol Chem 272:7873–7882. 10.1074/jbc.272.12.7873 9065454 10.1074/jbc.272.12.7873

[50] Otmakhov N, Khibnik L, Otmakhova N et al (2004) Forskolin-Induced LTP in the CA1 hippocampal region is NMDA receptor dependent. J Neurophysiol 91:1955–1962. 10.1152/jn.00941.2003 14702333 10.1152/jn.00941.2003

[51] Otsu M, Hiles I, Gout I et al (1991) Characterization of two 85 kd proteins that associate with receptor tyrosine kinases, middle-T/pp60c-src complexes, and PI3-kinase. Cell 65:91–104. 10.1016/0092-8674(91)90411-q 1707345 10.1016/0092-8674(91)90411-q

[52] Welch HCE, Coadwell WJ, Stephens LR, Hawkins PT (2003) Phosphoinositide 3-kinase-dependent activation of Rac. FEBS Lett 546:93–97. 10.1016/s0014-5793(03)00454-x 12829242 10.1016/s0014-5793(03)00454-x

[53] Heffetz D, Bushkin I, Dror R, Zick Y (1990) The insulinomimetic agents H2O2 and vanadate stimulate protein tyrosine phosphorylation in intact cells. J Biol Chem 265:2896–2902 2154464 10.1016/S0021-9258(19)39885-0

[54] Ito Y, Vogt PK, Hart JR (2017) Domain analysis reveals striking functional differences between the regulatory subunits of phosphatidylinositol 3-kinase (PI3K), p85α and p85β. Oncotarget 8:55863–55876. 10.18632/oncotarget.19866 28915558 10.18632/oncotarget.19866 PMC5593529

[55] Vallejo-Díaz J, Chagoyen M, Olazabal-Morán M et al (2019) The opposing roles of PIK3R1/p85α and PIK3R2/p85β in Cancer. Trends cancer 5:233–244. 10.1016/j.trecan.2019.02.009 30961830 10.1016/j.trecan.2019.02.009

[56] Luo J, Sobkiw CL, Logsdon NM et al (2005) Modulation of epithelial neoplasia and lymphoid hyperplasia in PTEN+/- mice by the p85 regulatory subunits of phosphoinositide 3-kinase. Proc Natl Acad Sci U S A 102:10238–10243. 10.1073/pnas.0504378102 16006513 10.1073/pnas.0504378102 PMC1174923

[57] Cortés I, Sánchez-Ruíz J, Zuluaga S et al (2012) p85β phosphoinositide 3-kinase subunit regulates tumor progression. Proc Natl Acad Sci U S A 109:11318–11323. 10.1073/pnas.1118138109 22733740 10.1073/pnas.1118138109 PMC3396516

[58] Ito Y, Hart JR, Ueno L, Vogt PK (2014) Oncogenic activity of the regulatory subunit p85β of phosphatidylinositol 3-kinase (PI3K). Proc Natl Acad Sci U S A 111:16826–16829. 10.1073/pnas.1420281111 25385636 10.1073/pnas.1420281111 PMC4250105

[59] Arendt KL, Benoist M, Lario A et al (2014) PTEN counteracts PIP3 upregulation in spines during NMDA-receptor-dependent long-term depression. J Cell Sci 127:5253–5260. 10.1242/jcs.156554 25335889 10.1242/jcs.156554

[60] Tolias KF, Bikoff JB, Burette A et al (2005) The Rac1-GEF Tiam1 couples the NMDA receptor to the activity-dependent development of dendritic arbors and spines. Neuron 45:525–538. 10.1016/j.neuron.2005.01.024 15721239 10.1016/j.neuron.2005.01.024

[61] Saneyoshi T, Matsuno H, Suzuki A et al (2019) Reciprocal activation within a kinase-effector Complex underlying persistence of structural LTP. Neuron 102:1199–1210e6. 10.1016/j.neuron.2019.04.012 31078368 10.1016/j.neuron.2019.04.012 PMC6669903

[62] Elam WA, Cao W, Kang H et al (2017) Phosphomimetic S3D cofilin binds but only weakly severs actin filaments. J Biol Chem 292:19565–19579. 10.1074/jbc.M117.808378 28939776 10.1074/jbc.M117.808378 PMC5712599

[63] Kuhn TB, Meberg PJ, Brown MD et al (2000) Regulating actin dynamics in neuronal growth cones by ADF/cofilin and rho family GTPases. J Neurobiol 44:126–144 10934317 10.1002/1097-4695(200008)44:2<126::AID-NEU4>3.0.CO;2-Z

[64] Dwivedi Y, Rizavi HS, Teppen T et al (2008) Lower phosphoinositide 3-kinase (PI 3-kinase) activity and differential expression levels of selective catalytic and regulatory PI 3-kinase subunit isoforms in prefrontal cortex and hippocampus of suicide subjects. Neuropsychopharmacol off Publ Am Coll Neuropsychopharmacol 33:2324–2340. 10.1038/sj.npp.1301641 10.1038/sj.npp.1301641 18075493

[65] Okamoto T, Namikawa K, Asano T et al (2004) Differential regulation of the regulatory subunits for phosphatidylinositol 3-kinase in response to motor nerve injury. Brain Res Mol Brain Res 131:119–125. 10.1016/j.molbrainres.2004.08.015 15530660 10.1016/j.molbrainres.2004.08.015

[66] Moreno-Corona N, Chentout L, Poggi L et al (2021) Two Monogenetic disorders, activated PI3-Kinase-δ syndrome 2 and Smith-Magenis Syndrome, in one patient: Case Report and a literature review of neurodevelopmental impact in primary immunodeficiencies Associated with disturbed PI3K signaling. Front Pediatr 9:688022 34249818 10.3389/fped.2021.688022 PMC8266209

[67] Fuller L, Dailey ME (2007) Preparation of rodent hippocampal slice cultures. CSH Protoc 2007:pdb.prot4848. 10.1101/PDB.PROT4848 10.1101/pdb.prot4848 21356949

[68] Gähwiler BH, Capogna M, Debanne D et al (1997) Organotypic slice cultures: a technique has come of age. Trends Neurosci 20:471–477. 10.1016/S0166-2236(97)01122-3 9347615 10.1016/S0166-2236(97)01122-3

[69] Banker GA, Cowan WM (1977) Rat hippocampal neurons in dispersed cell culture. Brain Res 126:342–397. 10.1016/0006-8993(77)90594-7 10.1016/0006-8993(77)90594-7 861729

[70] Fernández-Monreal M, Sánchez-Castillo C, Esteban JA (2016) APPL1 gates long-term potentiation through its plekstrin homology domain. J Cell Sci 129:2793–2803. 10.1242/jcs.183475 27257087 10.1242/jcs.183475

[71] Chen HX, Otmakhov N, Lisman J (1999) Requirements for LTP induction by pairing in hippocampal CA1 pyramidal cells. J Neurophysiol 82:526–532. 10.1038/361031a0 10444652 10.1038/361031a0

[72] Huber KM, Kayser MS, Bear MF (2000) Role for rapid dendritic protein synthesis in hippocampal mGluR- dependent long-term depression. Sci (80-) 288:1254–1256. 10.1126/science.288.5469.1254 10.1126/science.288.5469.1254 10818003

