# Supplementary material for: PI3K couples long-term synaptic potentiation with cofilin recruitment and actin polymerization in dendritic spines via its regulatory subunit p85α
Source: Cell Mol Life Sci. 2024 Aug 19;81(1):358. doi: 10.1007/s00018-024-05394-x (PMC11335278; doi:10.1007/s00018-024-05394-x)
Supplement: Supplementary file 1 — Supplementary Material 1 [file 18_2024_5394_MOESM1_ESM.pdf]

SUPPLEMENTAL MATERIAL

Supplementary Figure 1. Specificity and efficacy of p85 shRNAs. A. Schematic representation of the domain organization of PI3K regulatory subunits (p85α, p85β, p50α/p55α) and their association with the PI3K catalytic subunit (p110) and Rac1/Cdc42 small GTPases. **B.** *Left*: Representative western blot of whole protein extracts from dissociated primary hippocampal neurons infected with lentiviruses expressing shp85α (L+S), shp85α (L), shp85β or an empty vector (EV, control) coupled to mCherry as a reporter. Membranes were developed with specific antibodies for p85α, p85β, p110α, p110β, mCherry (control for infection levels) and actin (loading control), as indicated. *Right*: Quantification of the protein levels detected in A. Protein levels are normalized to the loading control and then to the mean protein levels from the empty vector-infected samples. Individual values for each experiment are represented as lines overlying the columns. Bars show mean±SEM. n: number of independent experiments. Statistic was assessed by Wilcoxon t-test (*). Comparisons were made against the empty vector. ****p<0.0001, *p<0.05, ns: not significant. **C.** *Left*. Representative Western blots of protein extracts from primary hippocampal neurons infected with lentivirus expressing shp85α, shp85β or an empty vector (EV, control), and immunoprecipitated using an anti-p110α antibody. Non-immune rabbit (Rb) IgG's were used as immunoprecipitation control. Protein levels are shown for bound and unbound fractions using specific antibodies for p85α, p85β and p110α. Relevant comparisons are highlighted with green (p85α co-precipitation) and blue (p85β co-precipitation) rectangles. *Right*. Quantification of immunoprecipitated protein levels expressed as the percentage of total protein. Individual values for each experiment are represented as lines

overlying the columns. Bars represent mean±SEM. IP: antibody used for immunoprecipitation.

Supplementary Figure 2. Passive membrane properties and spine density in shRNA-expressing neurons and treated with PI3K o NMDAR inhibitors. **A.** Input resistance (left) and whole-membrane capacitance (right) of CA1 pyramidal neurons from organotypic hippocampal slices infected with lentiviruses expressing shp85α (L+S), shp85α (L), shp85β or uninfected controls. Individual values for each cell are represented as lines overlying the columns. Bars show mean±SEM. No statistically significant difference was found with the Kruskal-Wallis test. **B.** Spine density of CA1 pyramidal neurons from hippocampal organotypic slices biolistically co-transfected with cofilin-GFP together with empty vector (mCherry control) or with vectors for the expression of shp85α (L+S), shp85α (L), shp85β, BHα or BHβ. Some slices were also treated with APV, LY294002 or vehicle control (DMSO). Spine density was calculated as the number of spines divided by the length of the dendritic segment. Individual values for each experiment are represented as lines overlying the columns. Bars represent mean±SEM. No statistically significant difference was found with the Kruskal-Wallis test.

Supplementary Figure 3. Analysis of PI3K activity and Rac1 activation by peroxovanadate treatment. **A.** Pull-down of active (GTP-bound) Rac1 or Cdc42 with GST-PAK-PBD from extracts of hippocampal neurons. Extracts were incubated with GDP or with the GTP analog GMP-PNP, as indicated. GST alone was used as negative control. Bound and input fractions were analyzed by

Western blot using antibodies specific for Rac1 or Cdc42. Lower blot shows GST protein expression. **B.** Western blot analysis of PI3K activity (as monitored by Akt phosphorylation) from extracts of primary hippocampal neurons expressing shp85α (L+S), shp85β or an empty vector (EV, control), and treated or not with peroxovanadate (pV). Blots for p85α and p85β served to test the efficiency of the shRNAs. Actin was used as loading control, and mCherry as control for infection levels. **C.** Quantification of P-Akt T308 levels, from experiments as the one shown in B. Individual values for each experiment are represented as lines overlying the columns. Bars represent mean±SEM. n: number of independent experiments. Significant differences between conditions were assessed by Mann-Whitney U test. ***$p<0.001$, **$p<0.01$, *$p<0.05$.

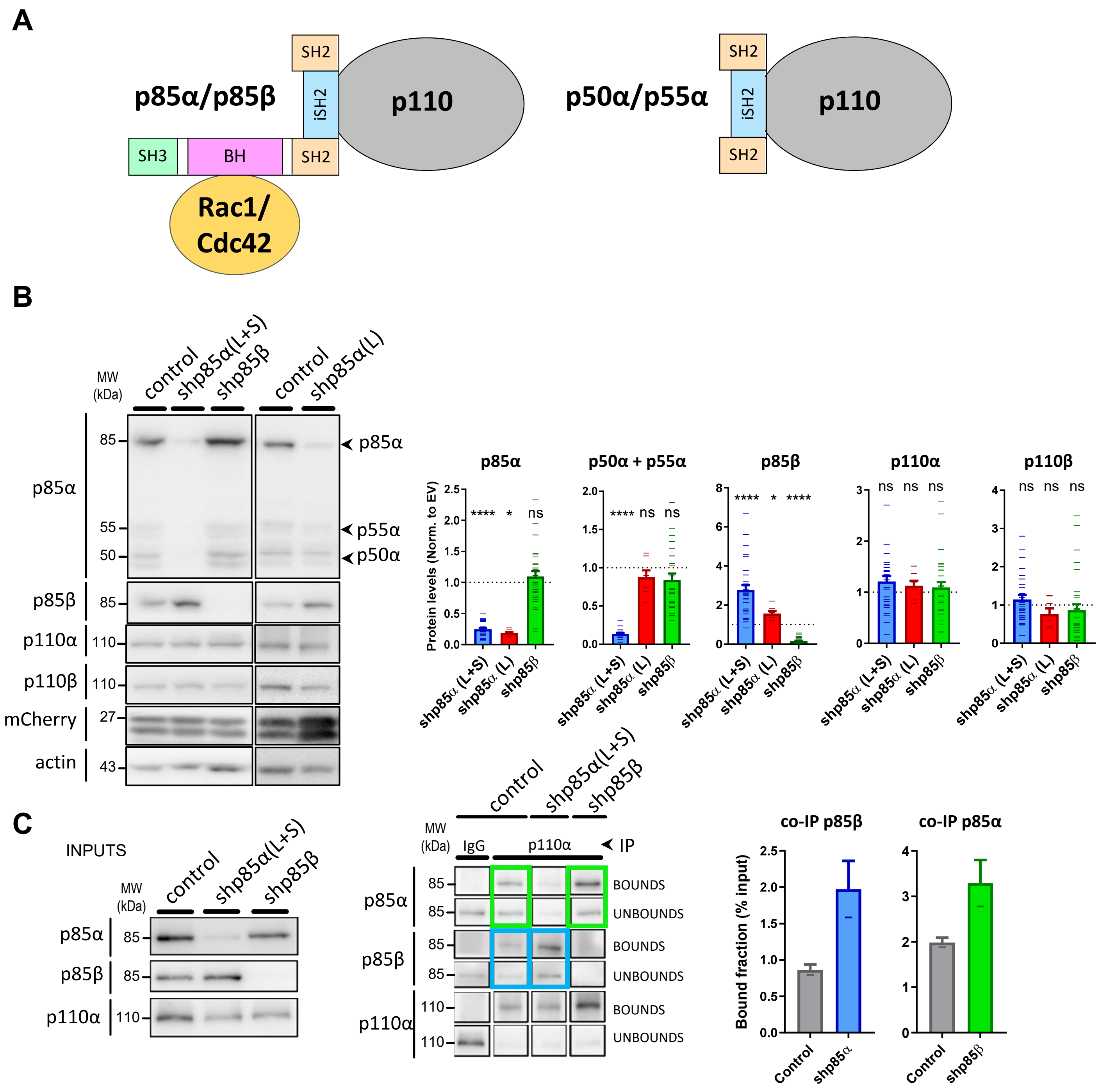

Supplementary Figure 1

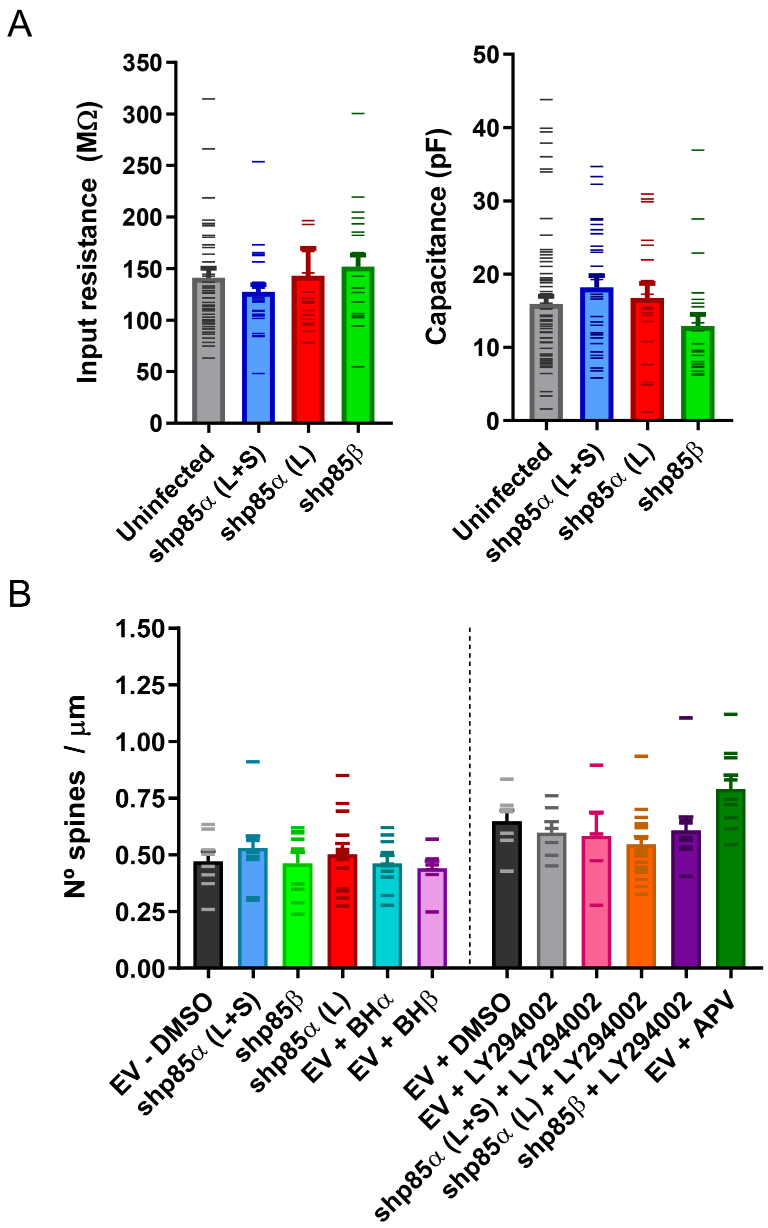

Supplementary Figure 2

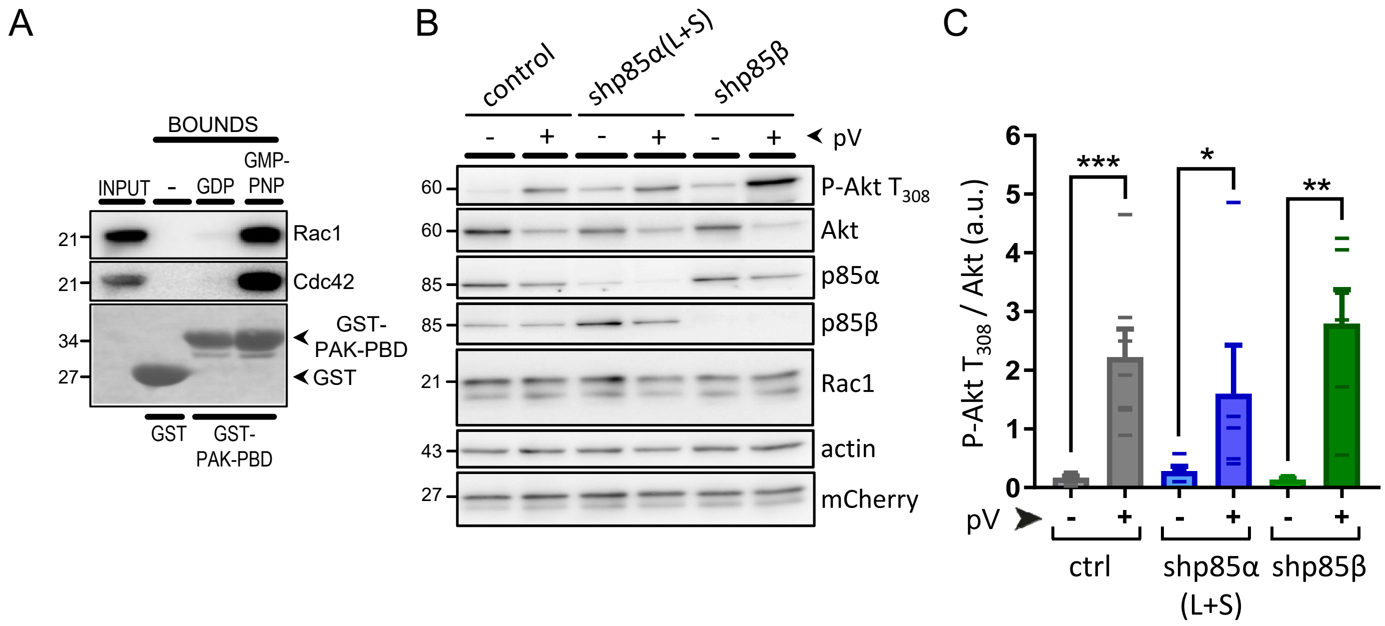

Supplementary Figure 3
